# Fishy Cooperation

**DOI:** 10.1371/journal.pbio.0040444

**Published:** 2006-12-05

**Authors:** Frans B. M. de Waal

It is commonly thought that animals can be arranged along a ladder of intelligence—a sort of modern-day *Scala Naturae*—with humans inevitably at the top, followed by our close relatives, the primates, all the way down to fish and other slimy creatures.

Over the past decade, this ladder has been challenged by claims of high intelligence and great social complexity in other animals. For example, spotted hyenas *(Crocuta crocuta)* establish hierarchies in which dominant females support the rank contests of their daughters. Bottlenose dolphins *(Tursiops aduncus)* form “political” coalitions every bit as complex as those of chimpanzees. Caledonian crows *(Corvus moneduloides)* not only use tools in the wild, but also modify tools in the lab, an ability once thought to define humans.

And now come the fish. It started with a provocative challenge to primate supremacy with the claim that “culture” (that is, socially transmitted behavior) is at least as well developed in fish as it is in primates. While this may be a bit of an exaggeration, a new study on cooperative behavior by Redouan Bshary and his colleagues really makes one wonder if there is anything fish cannot do.

The article describes the astonishing discovery of coordinated hunting between groupers *(Plectropomus pessuliferus)* and giant moray eels *(Gymnothorax javanicus)* in the Red Sea. These two species make a perfectly complementary pair. The moray eel can enter crevices in the coral reef, whereas the grouper hunts in open waters around the reef. Prey can escape from the grouper by hiding in a crevice and from the moray eel by leaving the reef, but prey has nowhere to go if hunted by a combination of these two predators.

The article offers a description and accompanying videos, such as the one showing a grouper and eel swimming side by side as if they are good friends on a stroll. It also offers quantification, which is truly hard to achieve in the field, of the tendencies involved in this mutually beneficial arrangement. The investigators were able to demonstrate that the two predators seek each other’s company, spending more time together than expected by chance. They also found that groupers actively recruit moray eels through a curious head shake made close to the moray eel’s head to which the eel responds by leaving its crevice and joining the grouper. Groupers showed such recruitment more often when hungry.

Given that cooperative hunting increases capture success for each of the two predators, and that they don’t share with each other but swallow the prey whole, their behavior seems a form of “by-product mutualism,” defined as a form of cooperation in which both parties achieve rewards without sacrificing anything for the other. They are both out for their own gain, which they attain more easily together than alone.

The observed role division comes “naturally” to two predators with different hunting specializations, and is therefore far simpler to achieve than for members of the same species. Also, recruitment is quite common in the animal kingdom—for example, primates have specialized signals to solicit each other’s support in fights. What is truly spectacular about this study is that the entire interaction pattern—two actors who seemingly know what they are going to do and how this will benefit them—is not one we usually associate with fish. This is probably because we tend to develop cognitively demanding accounts for our own behavior and believe that absent the same cognition, the behavior simply cannot take place. It is very well possible, however, that our accounts overestimate the amount of intelligence that goes into complex behavior. Moreover, we have a tendency to underestimate the intelligence of animals at lower rungs of the evolutionary ladder.

In fact, it is the ladder idea itself that is wrong. The best way to approach animal intelligence is from an evolutionary and ecological perspective focused on the tasks that each species faces in nature. In this regard, these two reef predators show us that if it comes to survival, highly intelligent solutions are within the reach of animals as different from us as fish. (Watch a grouper signal to a giant moray eel resting in a cave by shaking its head in front of the moray in this video. DOI: 10.1371/journal.pbio.0040431.sv001)

